# Need for cognition and burnout in teachers – A replication and extension study

**DOI:** 10.1177/20551029221139679

**Published:** 2022-11-11

**Authors:** Josephine Zerna, Nicole Engelmann, Anja Strobel, Alexander Strobel

**Affiliations:** 1 Faculty of Psychology, 9169 Technische Universität Dresden, Dresden, Germany; 2 Faculty of Education, 9169 Technische Universität Dresden, Dresden, Germany; 3 Institute of Psychology, 38869 Chemnitz University of Technology, Chemnitz, Germany

**Keywords:** burnout, behaviour, resources, demands, education, Covid-19

## Abstract

Burnout has become more prevalent, mainly in social jobs, and there is evidence that certain personality traits protect against burnout. Only recently, studies have focused on investment traits like Need for Cognition (NFC), the stable intrinsic motivation to seek out and enjoy effortful cognitive activities. This study had three aims: First, the replication of findings by Grass et al. (2018), who investigated NFC and the burnout subscale reduced personal efficacy in student teachers, in a sample of 180 teachers. Second, investigating the role of perceived demands and resources in the context of NFC and burnout. And finally, creating an exploratory model for further research. The results indicated that unlike the student sample, the teachers’ association of NFC and reduced personal efficacy was mediated by self-control but not reappraisal. Teachers with higher NFC and self-control also had lower burnout because they experienced their resources as fitting to the demands.

## Introduction

Need for Cognition (NFC) is a stable intrinsic motivation to seek out and especially to enjoy effortful cognitive activities (Cacioppo and Petty, 1982). As it bridges the gap between cognition and motivation, NFC is considered to be an investment trait (Stumm and Ackerman, 2013), and has come to the fore of psychological research in the last years. NFC can easily be assessed using the Need for Cognition Scale (NCS), a self-report questionnaire with 18–34 items (Cacioppo et al., 1984; Cacioppo and Petty, 1982; German form: Bless et al., 1994). While many studies have found medium sized positive associations of NFC with academic performance (Cazan and Indreica, 2014; Grass et al., 2017; Lavrijsen et al., 2021; Stumm and Ackerman, 2013; Zheng et al., 2020), recent investigations have also looked at NFC as a personal resource in academic and work contexts. Individuals high in NFC have more positive emotions at the end of the work day (Rosen et al., 2020), higher work motivation, perceive their roles as less ambiguous (Nowlin et al., 2017), are less likely to drop out of college (Grass et al., 2017; Klaczynski and Fauth, 1996), and have less anxiety regarding their course work (Karagiannopoulou et al., 2020), with these associations being in a small to medium range. These findings suggest that individuals high in NFC might be less prone to experience adverse effects of work stress, which range from physical (Dragano et al., 2017; Steptoe and Kivimäki, 2013) to psychological consequences (Madsen et al., 2017; Maslach and Leiter, 2016; Wiesner et al., 2005).

One of these psychological consequences is burnout, a state of exhaustion and cynicism caused by long-term overstimulation in the workplace, which results in employees being dissatisfied, being sick more often, and performing poorly (Schaufeli and Salanova, 2014). Burnout is especially prevalent in social jobs such as healthcare or teaching because the worker is often in conflict between advocating for their client and meeting the goals set by the employer (Gray-Stanley and Muramatsu, 2011; Lloyd et al., 2002). Lackritz (2004) found that university teachers’ burnout scores were higher the more students they had, the higher their teaching load was, and the more time they spent grading students’ work. Burnout is most often assessed using the Maslach Burnout Inventory (MBI) (Maslach et al., 1997), a self-report questionnaire with three subscales: *Emotional exhaustion*, a sense of fatigue and depletion, *depersonalisation*, a negative attitude towards clients, along with a loss of idealism, and *reduced personal efficacy*, a perceived decline of capability and coping skills.

### Replication of findings in student teachers in a teacher sample

Individuals with high burnout scores are often passive copers, high in neuroticism, low in self-esteem, and have an external locus of control (Schaufeli and Salanova, 2014). NFC on the other hand shows opposite associations with those variables (*r* = −0.19 with passive coping (Grass et al., 2018), *r* = −0.19 to −0.33 with neuroticism (Fleischhauer et al., 2019), *r* = 0.42 with self-esteem (Osberg, 1987), *r* = 0.32 with internal locus of control (Fletcher et al., 1986)), suggesting that people high in NFC are less prone to experience burnout. This is supported by findings that NFC showed moderate to large negative associations with burnout scores in adults (Fleischhauer et al., 2019), students (Fleischhauer et al., 2019; Naderi et al., 2018), and student teachers (Grass et al., 2018). However, the associations of NFC with the sum score and the subscales of the MBI are not always consistent between these studies. This is likely not caused by inaccurate measurement, since the validity of both NCS (Bless et al., 1994; Osberg, 1987; Tolentino et al., 1990) and MBI (Brady et al., 2021; Kantas and Vassilaki, 1997; Schaufeli et al., 2001; Valdivia Vázquez et al., 2021) has been demonstrated in multiple studies. What is more likely is the influence of one or more other variables, moderating or mediating the association of NFC and burnout. Grass et al. (2018) investigated such a mediation in a sample of 167 students, who were studying to become teachers in primary, secondary, special needs, or vocational schools, and who had been studying for *M* ± *SD* = 5.13 ± 2.63 semesters. They found that the relation of NFC and the MBI subscale *reduced personal-efficacy* was fully mediated by higher habitual use of reappraisal, more active coping, and less passive coping, but not by habitual use of suppression or self-control. Reappraisal and suppression are two emotion regulation strategies, which refer to the cognitive reassessment of a stressor and the inhibition of emotional reactions, respectively (Gross, 1998). The findings by Grass et al. (2018) suggest that individuals high in NFC experience a weaker decline in personal efficacy in response to long-term stress because they actively reassess the situation in a way that reinforces their sense of self-efficacy and don’t avoid dealing with the stressor (Strobel et al., 2017). One goal of this paper was to replicate the findings of Grass et al. (2018) in a teacher sample using a multiple mediation model on cross-sectional self-report data of teachers. We hypothesized that NFC would be negatively associated with the MBI subscale *reduced personal efficacy* via higher reappraisal scores, but not via suppression, via self-control, or directly, respectively.

### Demand-resource-model

Furthermore, we extended the analysis to other possible mediators. These mediators were motivated by our own recent survey of the literature on NFC and well-being, which suggested that individuals high in NFC might not only have a high level of personal resources but also overestimate their own resources to a certain degree (Zerna et al., 2021). This can take the form of patients with higher NFC having lower intentions to consult their doctor (Latimer et al., 2007) or adults with higher NFC having higher intentions to exhibit a harmful behavior after an intervention (Gallivan, 2020). Only a balance of resources and demands results in personal well-being, while an imbalance threatens well-being, regardless of whether this imbalance is in favour of resources or demands (Dodge et al., 2012). Following the framework of Hobfoll (1989), resources can be objects with practical or status purpose, conditions like marriage or tenure, personality aspects like coping style, and energies such as time, money, or knowledge. In the case of NFC, resources are from the categories personality and energies: Personality, because NFC is a trait, encompassing a curious, analytic, and passionate approach to challenges, and energies, because individuals high in NFC have been coping actively all their life, which enriches their level of experience and knowledge in approaching challenges (Cacioppo et al., 1996). These personal resources matter with regard to stress assessment and with regard to both coping and recovery (Salanova et al., 2006). We therefore investigated whether the association of NFC and burnout was mediated by different ratios of demands and resources; demands that are too high to be dealt with using one’s personal resources (*demands too high*), demands that are too low for one’s personal resources (*demands too low*), and a balanced fit of demands and resources (*demand-resource-fit*). Using the same data as for the replication, we computed a structural equation model (SEM) to assess the influence of these mediators. Since individuals high in NFC are confident in their abilities (Bye and Pushkar, 2009; Ghorbani et al., 2004; Heppner et al., 1983; Klaczynski and Fauth, 1996), we hypothesized that NFC would be negatively associated with *demands too high*, and positively associated with *demands too low* and *demand-resource-fit*, respectively. And since burnout results from constant unpleasant activation by high demands, we hypothesized that the MBI score would be positively associated with *demands too high* and negatively associated with *demand-resource-fit*, respectively. However, we had no hypothesis regarding the association of *demands too low* and the MBI score. Even though *demands too low* is akin to the concept of boredom and the consequences of boredom and burnout are very similar, burnout is a state with even lower activation and even more negative affect than boredom (Schaufeli and Salanova, 2014).

It has already been shown that the Covid-19 pandemic has exacerbated the rising prevalence of burnout (Fröbe and Franco, 2021), so we incorporated the degree of feeling burdened by the pandemic in an exploratory approach.

To sum up, this study used one questionnaire data set to investigate three aims: Firstly, replicating findings of a mediation between NFC and *reduced personal efficacy* in a teacher sample to identify possible differences between teachers and student teachers. Secondly, investigating the impact of different demand-resource-ratios on the relationship between NFC and burnout to see whether teachers with high NFC tend to overestimate their own resources or underestimate the current demands, respectively. And lastly, exploring the impact of other variables in the burnout context, such as the perceived burden by the pandemic, to create a starting point for future research.

## Methods

We report how we determined our sample size, all data exclusions (if any), all manipulations, and all measures in the study (Simmons et al., 2012). Our preregistration, the data, and the analysis script are available at https://osf.io/36ep9/. The procedure was evaluated and approved by the Ethics Committee of the Chemnitz University of Technology (reference number: V-389-15-AS-Burnout-08062020). It was not considered to require further ethical approvals and hence, deemed uncritical concerning ethical aspects according to the criteria used by the Ethics Committee, which includes aspects of the sample of healthy adults, voluntary attendance, noninvasive measures, no deception, and appropriate physical and mental demands on the subject.

### Participants

The initial study was aiming to recruit a sample of *N ≥* 287 teachers as determined using the R package *pwr* (Champely, 2020), assuming a small to medium correlation of *r =* 0*.*20 between the measures of interest and targeting at a power of 1 *− β =* 0*.*80 at *α =* 0*.*01. Teachers were recruited via social media, personal contacts to teachers, and to Saxon schools with the request to pass on the information. All teachers were eligible, no payment was issued. Of the *N =* 278 participants, who started filling out the online survey, *N =* 180 (72.20% female, aged 20–67 years) data sets were complete and those participants indicated to have answered truthfully. All of them were currently teaching at a primary, secondary, comprehensive, or vocational school. Data was collected between the 12th of June and the 24th of July 2020. At this point, schools had been switching between digital and hybrid forms of teaching for at least 3 months due to the Covid-19 pandemic, causing additional stress for many teachers. This may have also be one reason why the original study did not reach the estimated sample size. The data had been collected for the thesis of N.E., which investigated the correlation of the MBI score with years of teaching experience, self-control, NFC, reappraisal, suppression, work satisfaction, and general wellbeing, as well as a mediation of NFC and the MBI score by self-control. The research questions of the present manuscript arose from a literature review on NFC and well-being by J.Z., An.S., and Al.S. (Zerna et al., 2021), which suggested that individuals high in NFC might overestimate their own resources. At the time of preregistering the present analyses, J.Z. and An.S. were not familiar with the results of the thesis, while N.E. and their supervisor Al.S. were. The raw data was given to J.Z., who wrote the preregistration.

For the present analyses, we used the smallest standardized indirect effect from the mediation in Grass et al. (2018) in a post hoc power analysis with G*Power (Faul et al., 2007, 2009), which yielded a power of 1 *− β =* 0*.*85 for linear multiple regression, given *α =* 0*.*05, *N =* 180, and *f*^2^ = 0*.*05.

### Materials

All questionnaires were used in their German form. Burnout was assessed using the 21-item Maslach Burnout Inventory (MBI, Büssing and Perrar, 1992), with items such as “I don’t really care what happens to some recipients” and responses on a 6-point Likert scale from 1 (a few times a year) to 6 (every day). Its subscales *emotional exhaustion*, *depersonalization*, and *reduced personal efficacy* showed acceptable internal consistency (Cronbach’s *α >* 0*.*70) and low retest reliabilities of *r*_*tt*_ *=* 0*.*60, *r*_*tt*_ *=* 0*.*54, and *r*_*tt*_ *=* 0*.*57, respectively, over the span of 1 year in teachers (Maslach et al., 1997). Following Dreher et al. (2019), *emotional exhaustion* scores of ≥22, *depersonalization* scores of ≥8, and *reduced personal efficacy* scores of ≥24 are considered high. NFC was assessed with the 16-item short version of the German NFC scale (NCS) (Bless et al., 1994), consisting of items (e.g. “Thinking is not my idea of fun,” recoded) that are answered on a 7-point Likert scale ranging from −3 (completely disagree) to +3 (completely agree). The scale shows comparably high internal consistency (Cronbach’s *α >* 0*.*80) (Bless et al., 1994; Fleischhauer et al., 2010) and a retest reliability of *r*_*tt*_ *=* 0*.*83 across 8–18 weeks (Fleischhauer et al., 2015). Self-control was measured using the short form of the German Self-Control Scale (SCS-K-D; Bertrams and Dickhäuser (2009)) that comprises 13 items (e.g. “I am able to work effectively toward long-term goals”) with a 5-point Likert scale ranging from −2 (completely disagree) to +2 (completely agree), with comparably high reliability (Cronbach’s *α >* 0*.*80, 7-week retest reliability *r*_*tt*_ *=* 0*.*82) (Bertrams and Dickhäuser, 2009). Reappraisal and suppression were measured using the 10-item Emotion Regulation Questionnaire (ERQ) (Abler and Kessler, 2009). The scale has items such as “When I’m faced with a stressful situation, I make myself think about it in a way that helps me stay calm” (reappraisal) and “I keep my emotions to myself” (suppression), which are answered on a 7-point Likert scale ranging from 1 (strongly disagree) to 7 (strongly agree), and has acceptable to high internal consistency (Cronbach’s *α >* 0*.*75) (Preece et al., 2019). Work satisfaction was assessed using the Allgemeine Arbeitszufriedenheit questionnaire (Fischer and Lück, 2014) with items such as “Sometimes I feel like my work doesn’t really matter in my firm,” which are answered on a 5-point Likert scale from 1 to 5 and different anchors depending on the item. The scale has a split-half reliability of *r*_*tt*_ *=* 0*.*95 (Fischer and Lück, 2014). Eleven items were created to assess each participant’s current burden by the Covid-19 pandemic, such as whether they belong to a risk group or whether they currently had a higher workload. The translated Covid-19 items can be found in the Supplementary Material S1. Due to a technical error during survey setup, the coping style data of the Erfurter Belastungsinventar (Böhm-Kasper et al., 2001) could not be used, so we could not replicate the mediation of NFC and burnout by active and passive coping.

### Procedure

The questionnaires were provided online using SoSci Survey (Leiner, 2019). Participants were informed about aims and duration of the study and data security, then they provided demographic information, answered the questionnaires, and could optionally enter their email address to be informed about the results of the analysis of N.E.’s thesis.

### Data analysis

We used *R Studio* (R Core Team, 2020, RStudio Team, 2020) with the main packages *lavaan* (Rosseel, 2012) and *psych* (Revelle, 2021) for all our analyses. Data were checked for multivariate normality using Mardia’s coefficient. To account for non-linear relationships, correlations were computed using Spearman’s rank coefficient rather than Pearson’s product moment correlation. Internal consistencies were assessed with Cronbach’s Alpha and MacDonald’s Omega. Since Cronbach’s Alpha has been criticized for being insensitive to violations of internal consistency (Dunn et al., 2014; Taber, 2018), the additional computation of MacDonald’s Omega had the purpose of ensuring a more reliable estimation. Correlations between variables were classified according the scheme by Gignac and Szodorai (2016), which recommends 0.10, 0.20, and 0.30 to be considered small, typical, and relatively large, respectively.

We did not include gender as a control variable. However, the gender ratio of the sample was very close to the ratio in all Saxon schools in the school year 2019/2020 (76.4% female) (Schule.sachsen.de., 2022).

#### Replication of Grass et al. (2018) in a teacher sample

Items were reverse coded according to the scale manuals. NFC and self-control were computed as the sum scores of the NCS and the SCS, respectively. *Reduced personal efficacy* was computed using the sum of the MBI subscale, and reappraisal and suppression were computed using the sum of each ERQ subscale. NFC was entered as the independent variable, having a direct and multiple indirect effects on MBI via self-control, reappraisal, and suppression as mediators. Following Grass et al. (2018), bootstrapped confidence intervals with *N =* 2000 replicates and the seed 13 were computed with the Bollen-Stine bootstrapping of *lavaan()* to account for deviations from normality. Multiple indices were used to evaluate model fit as recommended by Hu and Bentler (1999): The Chi-square test statistic, which measures the fit compared to a saturated model (small values indicate better fit of predicted and observed covariances), the Comparative Fit Index (CFI), which compares the fit to the baseline model (CFI* >* 0*.*95 indicates good fit, but CFI* >* 0*.*90 is also commonly used), the Standardized Root Mean Square Residual (SRMR), which compares the residuals of the observed and predicted covariance matrix (SRMR* <* 0*.*08 indicates fair fit, SRMR* <* 0*.*05 good fit), and the Root Mean Square Error of Approximation (RMSEA), which does the same as the latter but takes degrees of freedom and model complexity into account (RMSEA* <* 0*.*08 indicates acceptable fit, RMSEA* <* 0*.*05 good fit, RMSEA* <* 0*.*01 excellent fit).

#### Demand-resource-ratio model

All items, apart from those making up the demand-resource-ratios, were reverse coded according to the scale manuals. The latent factor NFC was computed by subjecting the NCS items to a parcelling procedure (Little et al., 2002), a method that is used in SEM when only relations between but not within constructs are of interest. Principal component analysis was used to determine the factor loadings of each NCS item onto the first component. Then, the items were randomly divided into four parcels and the average item loading per parcel was computed. This was repeated 10,000 times to find the parcelling choice with the smallest difference in average item loadings between parcels. The latent factor MBI was computed using the three subscales as indicators. For the demand-resource-ratios, we used three items from the work satisfaction scale each. The latent factor *demands too high* was indicated by items 4, 8, and 9, *demands too low* by the recoded items 12, 26, and 27, and *demand-resource-fit* by items 17, 22, and 24. The items can be translated as follows: 4) “There is too much pressure on me.”, 8) “There is often too much being demanded of us at work.”, 9) “I often feel tired and weary because of my work.”, 12) “I can realize my ideas here.”, 17) “I take pleasure in my work.”, 22) “Does your place of work give you the opportunity to do what you do best?”, 24) “Does your place of work give you enough opportunities to use your skills?”, 26) “Are you happy with your promotion prospects?”, and 27) “Are you happy with your position when comparing it to your skills?”. Model parameters were estimated using the maximum likelihood method with robust standard errors. Model fit was evaluated by looking at the Chi-square test statistic, CFI, SRMR, and RMSEA.

#### Exploratory analyses

We preregistered two exploratory analyses. Firstly, we repeated the SEM with the subscale *reduced personal efficacy* in place of the MBI score, since this subscale has shown higher correlations with NFC than the other subscales (Grass et al., 2018; Naderi et al., 2018). And secondly, we included a Covid-19 burden score into the SEM, computed as the sum of the Covid-19 items.

## Results

During visual inspection of correlation plots we noticed an unexpected outlier with very high MBI scores and very low NFC scores. A Q-Q-plot contrasting Mahalanobis *D*^*2*^ against expected Chi Square values confirmed the outlier. To adhere to the preregistration, we report the results containing the outlier in this section and the results excluding the outlier in the Supplementary Material S2.

### Descriptive statistics

Basic metric descriptives of the questionnaire scores and subscales are listed in Table 1. Only the ERQ sum score and its reappraisal subscale followed a normal distribution, so the results of the models should be interpreted with some caution and with a focus on indices that are robust against violation of normality, such as the Satorra-Bentler or Yuan-Bentler-scaled test statistics (Rosseel, 2012). Applying the cut-offs proposed by Dreher et al. (2019), this sample had high *emotional exhaustion* scores, high *depersonalization* scores, and low *reduced personal efficacy* scores.

**Table 1.** Descriptive statistics of the questionnaire scores.

Variable	Minimum	Maximum	Mean	SD	Normality	Skewness	Kurtosis
MBI	27	101	52.93	13.06	No	0.35	0.02
MBI EE	12	52	27.99	8.87	No	0.19	−0.59
MBI DP	5	24	9.72	3.26	No	0.82	0.86
MBI RPE	7	28	15.22	3.43	No	0.42	1.11
ERQ	16	63	39.18	7.82	Yes	−0.16	0.45
ERQ S	4	26	12.59	4.85	No	0.14	−0.73
ERQ R	9	42	26.59	6.29	Yes	−0.05	0.01
SCS	−19	23	7.79	8.42	No	−0.39	−0.22
NFC	−34	48	20.37	14.04	No	−0.59	0.56
DTH	−6	6	0.49	2.65	No	−0.15	−0.56
DTL	−6	6	−2.22	2.24	No	0.46	0.28
DRF	−4	6	3.63	1.79	No	−0.91	1.75
COV	14	33	24.53	4.28	No	−0.14	−0.70

*Note:* MBI: Maslach Burnout Inventory; MBI EE: Emotional exhaustion subscale; MBI DP: Depersonalisation subscale; MBI RPE: Reduced personal efficacy subscale; ERQ: Emotion Regulation Questionnaire; ERQ S: Suppression subscale; ERQ R: Reappraisal subscale; SCS: Self-Control Scale; NFC: Need for Cognition; DTH: Demands Too High; DTL: Demands Too Low; DRF: Demand-Resource-Fit; COV: Covid-19 Burden; SD: Standard deviation. *N =* 180.

Correlations and internal consistencies are displayed in Table 2. For this descriptive analysis, the variables *demands too high*, *demands too low*, and *demand-resource-fit* were computed as a sum of their item scores, not weighted as in the structural equation model. Using traditional cut-off values (Nunnally and Bernstein, 1994), the Cronbach’s Alpha of the three demand-resource-ratios can be considered acceptable. The more robust MacDonald’s Omega (Dunn et al., 2014) did not deviate much from Cronbach’s Alpha and indicated acceptable to good internal consistency. As expected, the MBI score showed a large positive correlation with *demands too high* (r_s_ = 0.67, *p* < 0.001) and a large negative one with *demand-resource-fit* (r_s_ = −0.55, *p* < 0.001). Surprisingly, the correlation between the MBI score and *demands too low* was positive and also large (r_s_ = 0.44, *p* < 0.001). The NFC score correlated negatively with the MBI sum score, and with all MBI subscales to a similar extent, contrary to some previous observations in other studies.

**Table 2.** Spearman correlations and internal consistencies of the questionnaire scores.

		1	2	3	4	5	6	7	8	9	10	11	12
1	MBI	0.90 (0.91)											
2	MBI EE	0.92***	0.91 (0.92)										
3	MBI DP	0.75***	0.54***	0.68 (0.69)									
4	MBI RPE	0.67***	0.43***	0.48***	0.79 (0.79)								
5	ERQ	−0.06	−0.06	0.04	−0.10	0.73 (0.62)							
6	ERQ S	0.05	0.00	0.17*	0.08	0.59***	0.75 (0.79)						
7	ERQ R	−0.10	−0.06	−0.06	−0.20**	0.71***	−0.07	0.84 (0.84)					
8	SCS	−0.34***	−0.28***	−0.37***	−0.19*	−0.03	−0.12	0.05	0.85 (0.86)				
9	NFC	−0.25***	−0.20**	−0.22**	−0.21**	−0.01	−0.18*	0.16*	0.22**	0.89 (0.89)			
10	DTH	0.67***	0.72***	0.35***	0.36***	0.03	0.05	−0.01	−0.21**	−0.15*	0.73 (0.73)		
11	DTL	0.44***	0.36***	0.38***	0.43***	0.01	0.16*	−0.14	−0.19*	−0.16*	0.41***	0.73 (0.75)	
12	DRF	−0.55***	−0.46***	−0.41***	−0.53***	0.00	−0.10	0.10	0.18*	0.24**	−0.42***	−0.56***	0.77 (0.77)
13	COV	0.24**	0.32***	0.08	0.02	−0.03	0.02	−0.07	−0.04	0.13	0.45***	0.10	−0.13

*Note*: MBI: Maslach Burnout Inventory; MBI EE: Emotional exhaustion subscale; MBI DP: Depersonalisation subscale; MBI RPE: Reduced personal efficacy subscale; ERQ: Emotion Regulation Questionnaire; ERQ S: Suppression subscale; ERQ R: Reappraisal subscale; SCS: Self-Control Scale; NFC: Need for Cognition; DTH: Demands Too High; DTL: Demands Too Low; DRF: Demand-Resource-Fit; COV: Covid-19 Burden. N = 180. * *p* < 0.05, ** *p* < 0.01, ** *p* < 0.001. Diagonal is Cronbach’s Alpha and (in brackets) MacDonald’s Omega. The diagonal value of Covid-19 Burden is Cronbach’s Alpha = 0.77 and MacDonald’s Omega = 0.81.

### Replication of Grass et al. (2018) in a teacher sample

**Figure 1.**
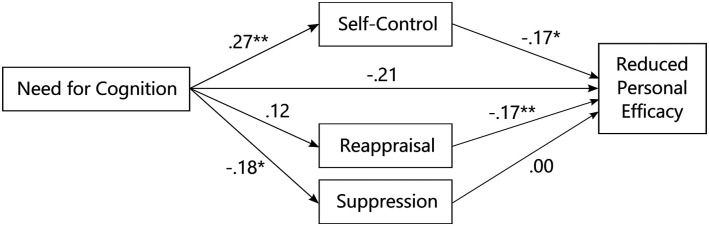
Standardized regression coefficients in the replication of Grass et al. (2018). *p < 0.05 and **p < 0.01.

In order to replicate findings by Grass et al. (2018) we computed a multiple mediation model with NFC as the independent variable, reduced personal efficacy as the dependent variable, and self-control, reappraisal, and suppression as mediators. The baseline model did not fit the data (*χ*^2^(10, *N* = 180) = 49.64, *p* < 0.001). Applying the cutoffs by Hu and Bentler (1999) to the fit indices of CFI = 1, TLI = 1.14, SRMR = 0.02, and RMSEA = 0.00, 95% CI [0.0.09], suggested good fit of the proposed model throughout all indices. Standardized estimates are displayed in Figure 1, total, direct, and indirect effects are listed in Table 3. We could replicate a positive association of NFC and self-control (β = 0.27, *p* = 0.002), and a negative association of habitual reappraisal and *reduced personal efficacy* (β = −0.17, *p* = 0.008). However, we could neither replicate the effect of NFC on reappraisal (β = 0.12, *p* = 0.105), nor the indirect effect of NFC on *reduced personal efficacy* via reappraisal (β = −0.02, *p* = 0.153). Furthermore, even though NFC and *reduced personal efficacy* were both associated with self-control in the correlational analysis, the indirect effect of NFC on *reduced personal efficacy* via self-control did not reach significance (β = −0.05, *p* = 0.090). Additionally, NFC was negatively associated with habitual use of suppression (β = −0.18, *p* = 0.012), which was not the case in the study by Grass et al. (2018).

**Table 3.** Results of the replication of Grass et al. (2018) in a teacher sample.

Path	*B*	*SE.*	*z*-value	*p*-value	CI lower	CI upper	*β*
Direct effects							
NFC on self-control	0.162	0.051	3.154	0.002	0.055	0.258	0.271
NFC on reappraisal	0.055	0.034	1.619	0.105	−0.011	0.120	0.123
NFC on suppression	−0.063	0.025	−2.524	0.012	−0.113	−0.017	−0.182
Self-control on RPE	−0.069	0.030	−2.318	0.020	−0.126	−0.009	−0.169
Reappraisal on RPE	−0.094	0.036	−2.652	0.008	−0.159	−0.023	−0.173
Suppression on RPE	0.002	0.051	0.043	0.966	−0.094	0.106	0.003
NFC on RPE	−0.051	0.021	−2.473	0.013	−0.089	−0.008	−0.208
Indirect effects							
NFC on RPE via self-control	−0.011	0.007	−1.695	0.090	−0.026	−0.001	−0.046
NFC on RPE via reappraisal	−0.005	0.004	−1.429	0.153	−0.013	0.001	−0.021
NFC on RPE via suppression	0.000	0.004	−0.039	0.969	−0.008	0.006	−0.001
Total effect							
Total effect	−0.067	0.023	−2.957	0.003	−0.111	−0.021	−0.276

*Note: B:* unstandardized regression coefficient; β: standardized regression coefficient; CI: confidence interval; NFC: Need for Cognition; RPE: reduced personal efficacy subscale of the Maslach Burnout Inventory; *SE:* standard error; *N* = 180.

Grass et al. (2018) controlled for age and a-level grade in their analysis, which we did not consider when preregistering this analysis. Since grade was not assessed in this sample, and age was assessed as a categorical variable, we instead incorporated how many years each participant had spent teaching at the point of assessment. We placed this variable as an independent variable influencing self-control, as the latter was the only variable in the model that showed a partial correlation with years spent teaching. As it was not preregistered, this was an exploratory analysis. Again, the baseline model did not fit the data (*χ*^2^(14, *N* = 180) = 60.41, *p* < 0.001), and the fit indices of CFI = 1, TLI = 1.19, SRMR = 0.02, and RMSEA = 0.00, 95% CI [0, 0.04], suggested good fit of the proposed model throughout all indices. Standardized estimates, total, direct, and indirect effects are displayed and listed in Supplementary Material S3. The associations between NFC, self-control, reappraisal, suppression, and *reduced personal efficacy* were almost identical to the first model. However, because of the positive association of years spent teaching and self-control (*β* = 0.22, *p* < 0.001), the indirect path leading from NFC and years spent teaching via self-control to *reduced personal efficacy* reached significance in this model (*β* = −0.09, *p* = 0.049). Therefore, the total effect also increased slightly, compared to the first model (*β* = −0.32, *p* = 0.002).

Following a reviewer’s request, we also analyzed this model with the *emotional exhaustion* and the *depersonalization* subscales as the outcome variable. The results are listed in Supplementary Table S6.1 and S6.2, respectively.

### Demand-resource model

**Figure 2.**
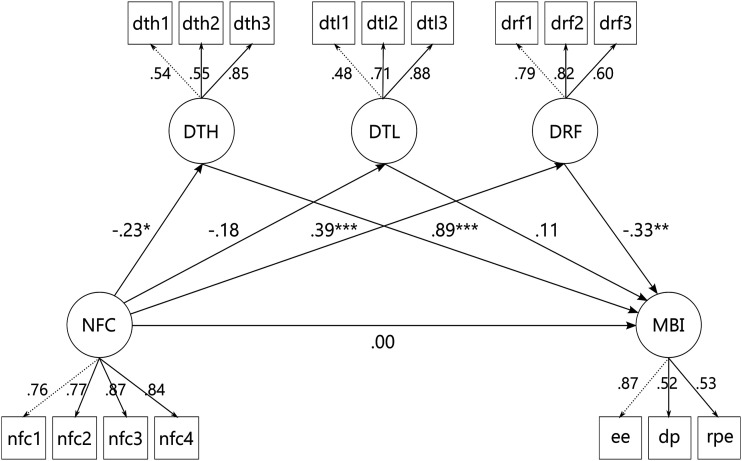
Standardized path coefficients in the mediation of NFC and burnout by demand-resource-ratios. *p < 0*.*05, **p < 0*.*01, ***p < 0*.*001. NFC: Need for Cognition; DTH: Demands Too High; DTL: Demands Too Low; DRF: Demand Resource Fit; MBI: Maslach Burnout Inventory; nfc1-4: item parcels; dth/dtl/drf1-3: item indicators; EE: Emotional Exhaustion; DP: Depersonalisation; RPE: Reduced Personal Efficacy.

Next we looked at how different ratios of subjective demands and resources affect the association of NFC and burnout. The parcelling procedure for the indicators of the latent factor NFC resulted in four parcels with a summed difference in average loadings of 0.00. The first parcel contained items 4, 6, 8, and 9, the second parcel items 2, 14, 15, and 16, the third parcel items 7, 11, 12, and 13, and the fourth parcel items 1, 3, 5, and 10. Standardized path coefficients of the demand-resource model are illustrated in Figure 2, total, direct, and indirect effects are listed in Table 4. The robust Chi-square statistic of *χ*^2^(97, *N* = 180) = 399.08 (*p <* 0*.*001) did not indicate good model fit. However, since it was in the range of 4 *df < χ*^2^ *>* 5 *df* the lack of good fit might have been due to the underlying assumption of multivariate normality (Hu and Bentler, 1999; Schumacker and Lomax, 2012), which was violated here. This also held true for the CFI of 0.78, the SRMR of 0.17, and the RMSEA of 0.13, 90% CI [0.12, 0.14]. Overall, the fit indices did not support the proposed model, and not all proposed paths were significant. NFC showed no direct association with the MBI score (*β =* 0.00, *p =* 0*.*989), even though it was negatively correlated with the sum score and all subscales. Instead, NFC showed indirect negative associations with the MBI score via lower scores in the latent variable *demands too high* (*β =* −0.20, *p =* 0*.*026) and via higher scores in the latent variable *demand-resource-fit* (*β =* −0.13, *p =* 0*.*025). The latent variable demands too low was neither related to NFC (*β =* −0.18, *p =* 0*.*128) nor to the MBI score (*β =* 0.11, *p =* 0*.*198).

**Table 4.** Results of the demand-resource-ratio model.

Path	*B*	*SE*	*z*-value	*p*-value	CI lower	CI upper	*β*
Direct effects							
NFC on DTH	−0.042	0.020	−2.154	0.031	−0.081	−0.004	−0.228
NFC on DTL	−0.023	0.015	−1.522	0.128	−0.052	0.007	−0.180
NFC on DRF	0.070	0.020	3.488	0.000	0.031	0.110	0.386
NFC on MBI	0.002	0.144	0.014	0.989	−0.281	0.285	0.001
DTH on MBI	10.624	2.229	4.767	0.000	6.256	14.991	0.892
DTL on MBI	1.838	1.428	1.287	0.198	−0.960	4.637	0.106
DRF on MBI	−4.036	1.080	−3.736	0.000	−6.153	−1.918	−0.332
Indirect effects							
NFC on MBI via DTH	−0.451	0.203	−2.221	0.026	−0.848	−0.053	−0.203
NFC on MBI via DTL	−0.042	0.033	−1.270	0.204	−0.107	0.023	−0.019
NFC on MBI via DRF	−0.284	0.127	−2.236	0.025	−0.533	−0.035	−0.128
Total effect							
Total effect	−0.775	0.258	−3.003	0.003	−1.280	−0.269	−0.349

*Note: B:* unstandardized regression coefficient; *β:* standardized regression coefficient; CI: confidence interval; DTH: Demands Too High; DTL: Demands Too Low; DRF: Demand Resource Fit; MBI: Maslach Burnout Inventory; NFC: Need for Cognition; *SE:* standard error.

### Exploratory analyses

The first exploratory analysis concerned a modification of the demand-resource-model in which the subscale *reduced personal efficacy* would be used in place of the MBI sum score. Path coefficients, total, direct, and indirect effects are displayed and listed in Supplementary Material S4. Similar to the previous model, this model’s indices did not indicate good fit, with a Chi-square statistic of *χ*^2^ = 247.82 (*p <* 0*.*001), a CFI of 0.83, an SRMR of 0.17, and an RMSEA of 0.12, 95% CI [0.10, 0.13]. NFC showed no direct association with *reduced personal efficacy* (*β =* −0.05, *p =* 0*.*551), but an indirect one via higher scores in the latent variable *demand-resource-fit* (*β =* −0.22, *p =* 0*.*002). And again, NFC was associated with lower scores in the latent variable *demands too high* (*β =* −0.22, *p =* 0*.*025), but the latter did not mediate the relationship between NFC and *reduced personal efficacy* (*β =* −0.03, *p =* 0*.*243) as it did with the MBI score in the previous model. The latent variable *demands too low* was neither related to NFC (*β =* −0.19, *p =* 0*.*102) nor to *reduced personal efficacy* (*β =* 0.11, *p =* 0*.*196).

Following a reviewer’s request, we also analyzed this model with the *emotional exhaustion* and the *depersonalization* subscales as the outcome variable. The results are listed in Supplementary Table S6.3 and S6.4, respectively.

**Figure 3.**
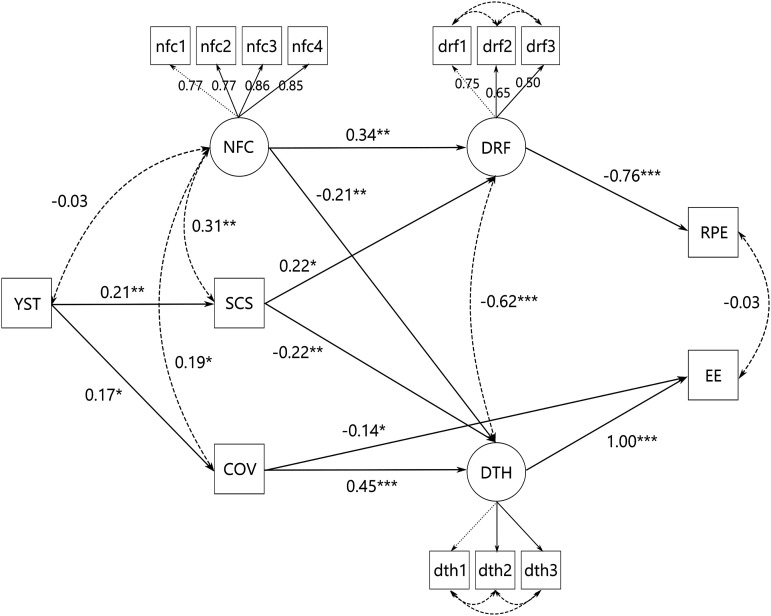
Standardized path coefficients in the exploratory analysis of variable relations. *p < 0*.*05, **p < 0*.*01, ***p < 0*.*001. YST: Years Spent Teaching; SCS: Self-Control Scale; COV: Covid Burden; NFC: Need for Cognition; DTH: Demands Too High; DRF: Demand Resource Fit; nfc1-4: Item Parcels; dth/drf1-3: Item Indicators; EE: Emotional Exhaustion; RPE: Reduced Personal Efficacy.

The second exploratory analysis concerned the incorporation of the Covid burden score into the model. We based the development of this model on the partial correlations of all variables, which provide an indication of how closely or remotely related variables might be in a path model. We started with a full model with all variables except for the ERQ and its subscales as they had hardly any correlations with the other variables. This model could not be computed by *lavaan*, so we modified the structure using the *modindices()*-function in order to increase the goodness-of-fit indices within the framework of contentually meaningful variable relationships. The final model is illustrated in Figure 3, the total, direct, and indirect effects are listed in Supplementary Material S5, along with the theorized full model’s structure. All fit indices suggested that the proposed model had good fit while the baseline model did not (*χ*^2^ = 130.13 (*p <* 0*.*001), CFI* =* 0.95, RMSEA* =* 0.07 (95% CI [0.05, 0.08]), *SRMR =* 0.06). Since the *depersonalisation* subscale of the MBI did not contribute significantly to the explained variance, it was not included in the final model. Years spent teaching was associated with higher self-control (*β =* 0.21, *p =* 0*.*002) and higher Covid burden (*β =* 0.17, *p =* 0*.*020) but not with NFC. NFC covaried with self-control (*σ =* 0.31, *p =* 0*.*008) and Covid burden (*σ =* 0.19, *p =* 0*.*018), but not with years spent teaching (*p =* 0*.*722). In turn, NFC was associated with higher *demand-resource-fit* scores (*β =* 0.34, *p =* 0*.*002) and lower *demands too high* scores (*β =* −0.21, *p =* 0*.*008) but not directly with any of the two MBI subscales. *Demand-resource-fit* scores fully mediated the negative association of NFC and self-control with *reduced personal efficacy* (indirect effect *β =* -0.29, *p =* 0.001), which was also true for *demands too high* scores and *emotional exhaustion*, but *demands too high* also partially mediated between Covid burden and *emotional exhaustion* (indirect effect *β =* −0.18, *p =* 0*.*008). Covid burden was not associated with *demand-resource-fit* or *reduced personal efficacy*.

## Discussion

The present study aimed to investigate the role of Need for Cognition, the stable preference for and enjoyment of cognitive effort, in the context of burnout in teachers. To achieve this, we replicated findings of mediators between Need for Cognition and *reduced personal efficacy*, and extended the analysis to the role of different ratios of demands and resources in burnout using latent variable models. In an exploratory approach, we investigated the influence of the burden that the Covid-19 pandemic has placed on teachers. Previous studies have indicated a protective effect of NFC against burnout, but the associations with the burnout subscales were inconsistent, suggesting that there are more variables influencing this relationship.

### Replication of Grass et al. (2018) in a teacher sample

While the mediation model had good fit, not all patterns were similar to the original study: NFC and self-control had a medium positive association, and reappraisal and *reduced personal efficacy* had a small negative association, but NFC and reappraisal were not associated. There was, however, a small negative association between self-control and *reduced personal efficacy*, and between NFC and suppression.

NFC had a direct, medium negative effect on *reduced personal efficacy*, but this relationship was not mediated by any other variable. Only when the amount of teaching experience was included as a predictor of self-control along with NFC, a very small indirect effect via self-control reached significance, indicating that teachers with high NFC and more years of teaching experience have higher self-control and, consequently, lower *reduced personal efficacy*. The higher self-control that comes with more teaching experience is in line with findings of fluctuations in self-control in young adults, reaching a low point between the age of 15 and 19 (Oliva et al., 2019). The participants in the study by Grass et al. (2018) were student teachers with a mean age of 25.5 years, while the majority of the current sample was between 40 and 59 years old. Therefore, it is likely that not only the teaching experience itself but also higher age might be associated with higher self-control. However, one could argue that more experience provides the teacher with a bigger repertoire of coping strategies to enable an efficient exertion of self-control, especially for teachers high in NFC who are intrinsically motivated to find and apply such strategies.

We could replicate the relation between the two emotion regulation strategies reappraisal and suppression with *reduced personal efficacy*, but not their association with NFC. There is ample evidence that reappraisal is associated with positive outcomes for students (Haga et al., 2007; Levine et al., 2012; Schmidt et al., 2010) and teachers alike (Jiang et al., 2016; Moè and Katz, 2020; Tsouloupas et al., 2010), so it is surprising that reappraisal did not mediate between NFC and *reduced personal efficacy*. Reappraisal did correlate with NFC, as it should appease the preference for cognitive effort in individuals with high NFC, but it was not a mediator in this model. One possible explanation could be that the ways by which reappraisal can be achieved, such as taking the role of an uninvolved observer, are less feasible for teachers in retaining their sense of efficacy in the classroom than the self-control needed to structurally manage students and situations. Hence, the mediation of NFC and *reduced personal efficacy* by self-control when taking the years spent teaching into account. Overall, the differences between Grass et al. (2018) and the present analysis suggest that burnout is being mediated by different factors in student teachers than in teachers. In a classroom context, controlling oneself and one’s emotional expression might be more relevant in dealing with the workload than in a university context, where a person is responsible for themself first and foremost. This could even imply a different interpretation of the concept of personal efficacy in these two samples––student teachers might focus on how well they can follow lectures, complete work on time, and schedule their tasks efficiently, while teachers might focus on how well they can manage their students and whether they get good grades. Therefore, the understanding of personal efficacy might change, encapsulating one’s own success or one’s students’ success depending on the career stage.

### Demand-resource-ratio model

Despite not having good fit indices, the model suggested a complete mediation of NFC and burnout via *demands too high* and *demand-resource-fit* but not *demands too low*. Specifically, individuals with higher NFC had lower burnout scores through perceiving demands as fitting to and not exceeding their own resources. Interestingly, the medium correlation between NFC and burnout disappeared in the context of the demand-resource-ratios as mediators. The mediator that did not reach significance was the perception of own resources exceeding the job demands. As this latent variable was conceptualized as boredom at work, we could not confirm the positive association of boredom and burnout found by Reijseger et al. (2013). However, the positive association of *demands too high* and *demands too low* in the correlation analysis suggests that boredom and burnout are not mutually exclusive. A teacher can have a very high workload, which is reflected in an elevated *demands too high* score, but the work in question might not be intellectually stimulating at all or might not appeal to their ideals and goals, which increases their *demands too low* score. Both of these variables were correlated with higher MBI scores, but in this model, high demands appear to have contributed more to explaining variance in MBI scores than low demands, suggesting that the perceived workload is more important in the burnout context than how stimulating the work is. The fact that the items that made up the demand-resource-ratios were about the subjective perception and not about objective measures, supports the idea that the individual appraisal of one’s own circumstances plays a crucial role in the development of burnout. This individual appraisal has been emphasized as the cause for the ambiguous impact of demands on psychological well-being before, in the form of challenge demands and hindrance demands (Lazarus and Folkman, 1984; Lepine et al., 2005; Podsakoff et al., 2007). Challenge demands such as time pressure, responsibility, and workload (Podsakoff et al., 2007) are being positively valued due to their potential to increase personal growth, positive affect, and problem-focused coping (Lepine et al., 2005). In contrast, hindrance demands such as inadequate resources, role conflict, and organizational politics (Podsakoff et al., 2007) are perceived as negative because they harm personal growth, trigger negative emotions, and increase passive coping (Lepine et al., 2005). Ventura et al. (2015) found that hindrance but not challenge demands were positively related to burnout in teachers, and teachers who reported high challenge and low hindrance demands also reported higher engagement. Whether and to what extent a circumstance is perceived as a challenge or hindrance demand is highly influenced by a person’s level of self-efficacy (Bandura, 1997), so much so that a reduction in self-efficacy is considered to be a precursor of burnout, not necessarily a symptom (Cherniss, 1993; Vera et al., 2012). Self-efficacy and self-control are closely entwined (Przepiórka et al., 2019; Vera et al., 2004; Yang et al., 2019) and both are positively associated with NFC (Bertrams and Dickhäuser, 2012; Holch and Marwood, 2020; Naderi et al., 2018; Xu and Cheng, 2021). Cacioppo et al. (1996) even proposed that higher levels of NFC might develop as a result of a high need for structure or control in those who have the skill, ability, and inclination to do so. These associations would imply that teachers with high levels of NFC report lower levels of burnout because their higher (desire for) self-control motivates them to appraise demands as a chance for personal growth, thereby meeting their passion for thinking and problem-solving. Nevertheless, appraisal is no universal remedy for circumstances that threaten well-being, as there certainly are circumstances that one cannot benefit from in any way. It remains an open question whether a high desire for control and high NFC might cloud one’s judgement in this case, by encouraging to invest one’s own insufficient resources in order to meet these high external demands. Such behavioural tendencies would threaten personal well-being in the long term, as the demands cannot be met, self-efficacy declines, and stress increases.

### Exploratory analyses

#### Demand-resource-ratio model with subscale reduced personal efficacy

The demand-resource-ratio model with the subscale *reduced personal efficacy* in place of the MBI score did not have good fit indices. Compared to the confirmatory demand-resource-ratio model, the mediation of NFC and *reduced personal efficacy* via *demands too high* did not reach significance, but both the mediation via *demand-resource-fit* and the total effect remained significant. Overall, this pattern did not resemble those from previous studies in which NFC had the strongest relation with this subscale of the MBI (Grass et al., 2018; Naderi et al., 2018). Teachers with high NFC appear to retain their sense of personal efficacy to a higher degree, because they experience a fit of demands and resources, which allows them to complete tasks and reinforce their sense of self-efficacy in return. However, while this association was similar in the confirmatory and the exploratory demand-resource-ratio model, the mediation via *demands too high* was not significant with this subscale, suggesting that the large association of *demands too high* and MBI in the confirmatory model was driven by a different subscale. To explore this, we built a second exploratory model.

#### Exploratory model with Covid burden

Due to the complete freedom in setting up the structure of this model, it had good fit indices. Interestingly, the third MBI subscale *depersonalisation* and the latent variable *demands too low* did not explain any variance in the model, so they were removed. Once again, NFC and self-control were positively related, but NFC was also positively related to Covid burden. One possible explanation is that teachers with higher NFC show higher consideration of the consequences and progression of the pandemic, thereby anticipating that it will take a long time until normal teaching can resume, which increases their feeling of being burdened. Although NFC has been shown to be related to more reflective thinking and unrelated to rumination, which are considered healthy and unhealthy thinking styles, respectively (Nishiguchi et al., 2018; Vannucci and Chiorri, 2018), a higher perceived Covid burden itself cannot indicate whether it stems from a realistic view on the pandemic or a feeling of being overwhelmed. Teachers with more years of experience also reported higher Covid burden, presumably because older people are less comfortable with technology (Hauk et al., 2018) and therefore stressed by the prospect of online teaching. Teachers with higher self-control and higher NFC reported a stronger fit of demands and resources, which was associated with a strong decrease in *reduced personal efficacy*. Higher self-control, higher NFC, and lower Covid burden was in turn associated with a lower *demands too high* score, so teachers with those characteristics felt less overwhelmed and consequently less emotionally exhausted. The degree of association between *demands too high* and *emotional exhaustion* indeed suggested a congruence between the two, indicating that *emotional exhaustion* in burnout is caused by excessive demands that cannot be met with one’s resources, while *reduced personal efficacy* in burnout is caused by a lack of opportunities to utilize one’s resources at work. Curiously, higher Covid burden also showed a small negative association with *emotional exhaustion*. It could be that for some teachers, remote teaching was experienced as a relief from the strain of dealing with a group of over 20 students each day, who are more likely to misbehave in a classroom setting than when they are studying at home. So while those teachers did feel the pandemic burden, they also felt less emotionally exhausted. However, as this part of the study was exploratory, the results should be interpreted with some caution and examined with new data in a confirmatory approach.

### Limitations and future implications

The data used in this study had been collected with a focus on emotion regulation and burnout, so there were several aspects that would have improved the investigation of our research questions but were not feasible. Firstly, collecting coping style data would have enabled a full replication of the mediation model of Grass et al. (2018). Secondly, longitudinal data would have facilitated more definitive conclusions about causal relations, as well as about inter-individual differences in the perception of demands and resources as the pandemic progresses. Furthermore, all of our data was self-report, which can decrease validity, but is not problematic when the perception is more predictive for the outcome than an objective measurement (Haeffel and Howard, 2010). And lastly, the latent variables for the demand-resource-ratios were item groups chosen from the work satisfaction questionnaire and had not been validated for this use before. However, as two of them showed meaningful relations with self-control, NFC, and two of the three MBI subscales, pursuing this concept further seems promising. Especially because we worked with pre-existing data, we preregistered all analyses and clearly differentiated between confirmatory and exploratory models in order to make the results as reliable as possible.

## Conclusions

Our study showed that self-control mediated between NFC and reduced personal efficacy when teaching experience was being taken into account. Contrary to prior studies, neither habitual use of reappraisal nor use of suppression mediated between NFC and burnout. However, a crucial role in the relation of NFC and burnout seemed to be the perceived ratio of personal resources and demands, specifically, a resource-demand-fit was associated with lower and excessive demands were associated with higher burnout scores. Applied to real-life teaching practice, our results suggest that a healthy work environment should offer ample opportunities to make use of one’s abilities, without creating demands that are too high. As a consequence, experiences and sense of self-efficacy will increase, which in turn heightens confidence in one’s skills to deal with future demands that are higher, preventing loss of personal efficacy and burnout in the long term.

## Supplemental Material

Supplemental Material - Need for cognition and burnout in teachers – A replication and extension study Click here for additional data file. Supplemental Material for Need for cognition and burnout in teachers – A replication and extension study by Josephine Zerna, Nicole Engelmann, Anja Strobel and Alexander Strobel in Health Psychology Open
