# Supplementary material for: Need for cognition and burnout in teachers – A replication and extension study
Source: Health Psychol Open. 2022 Nov 11;9(2):20551029221139679. doi: 10.1177/20551029221139679 (PMC9659943; doi:10.1177/20551029221139679)
Supplement: Supplemental Material - Need for cognition and burnout in teachers – A replication and extension study [file sj-pdf-1-hpo-10.1177_20551029221139679.pdf]

Supplementary Material

S1: Items and response scales used to assess Covid burden

1. How burdened do you currently feel by the measures associated with Covid-19?
    - [1] not burdened at all
    - [2] barely burdened
    - [3] rather burdened
    - [4] very burdened

2. Are you in a Covid-19 risk group? [*]
    - [1] yes
    - [2] no
    - [3] I don’t know

3. Do you have or have you had a Covid-19 infection? [*]
    - [1] yes
    - [2] no
    - [3] I don’t know

4. Are or were family members or other people close to you infected with Covid-19? [*]
    - [1] yes
    - [2] no
    - [3] I don’t know

5. Compared to before the pandemic, do you feel more burdened at work?
    - [1] no
    - [2] somewhat no
    - [3] somewhat yes
    - [4] yes

6. Compared to before the pandemic, are your worried more?
    - [1] no
    - [2] somewhat no
    - [3] somewhat yes
    - [4] yes

7. Compared to before the pandemic, do you feel restricted in your current day-to-day life?
    - [1] no
    - [2] somewhat no
    - [3] somewhat yes
    - [4] yes

8. Do you currently have additional responsibilities? [*]
    - [1] yes (which?)
    - [2] no

9. Compared to before the pandemic, how much time do you currently spend on leisure activities?
    - [1] less
    - [2] the same
    - [3] more

10. Compared to before the pandemic, do you currently spend more/less time on work-related activities (e.g. preparing lessons, reading literature, attending trainings for digital teaching)?
    - [1] less
    - [2] the same
    - [3] more

11. Compared to before the pandemic, how did the current demands within your job change?
    - [1] they are lower
    - [2] they are the same
    - [3] they are higher

[*] items with an asterisk are being reverse coded in the analysis, so that a higher score indicates a higher burden

S2: Results when excluding the outlier with very high MBI scores and very low NFC scores

Table S2.1

Spearman correlations and internal consistencies of the questionnaire scores.

		1	2	3	4	5	6	7	8	9	10	11	12
1	MBI	.90(.91)											
2	MBI EE	.92***	.91(.91)										
3	MBI DP	.74***	.53***	.68(.66)									
4	MBI RPE	.66***	.42***	.47***	.79(.79)								
5	ERQ	-.05	-.05	.05	-.10	.73(.62)							
6	ERQ S	.05	.00	.17*	.08	.59***	.75(.79)						
7	ERQ R	-.09	-.05	-.05	-.19*	.71***	-.07	.84(.84)					
8	SCS	-.33***	-.27***	-.36***	-.17*	-.04	-.12	.04	.85(.85)				
9	NFC	-.24***	-.18**	-.21**	-.20**	-.02	-.18*	.15*	.20**	.89(.88)			
10	DTH	.66***	.72***	.34***	.35***	.04	.05	.00	-.19**	-.13	.73(.72)		
11	DTL	.44***	.35***	.37***	.42***	.01	.16*	-.13	-.18*	-.15*	.40***	.73(.76)	
12	DRF	-.54***	-.45***	-.40***	-.52***	-.01	-.10	.09	.16*	.23**	-.41***	-.55***	.77(.75)
13	COV	.23**	.32***	.07	.00	-.02	.02	-.06	-.03	.14	.44***	.08	-.13

Note: MBI = Maslach Burnout Inventory, MBI EE = Emotional exhaustion subscale, MBI DP = Depersonalisationsubscale, MBI RPE = Reduced personal efficacy subscale, ERQ = Emotion Regulation Questionnaire, ERQ S = Suppression subscale, ERQ R = Reappraisal subscale, SCS = Self-Control Scale, NFC = Need for Cognition, DTH = Demands Too High, DTL = Demands Too Low, DRF = Demand-Resource-Fit, COV = Covid-19 Burden. N = 179. * p < .05, ** p < .01, *** p < .001. Diagonal is Cronbach's Alpha and (in brackets) MacDonald's Omega. The diagonal value of Covid-19 Burden is Cronbach's Alpha = .77 and MacDonald's Omega = .81.

Table S2.2

Results of the replication of Grass et al. (2018).

Path	*B*	*SE*	*z*-value	*p*-value	CI Lower	CI Upper	*β*
Direct Effects							
NFC on Self-Control	0.132	0.047	2.804	.005	0.042	0.226	0.217
NFC on Reappraisal	0.052	0.039	1.353	.176	-0.021	0.127	0.112
NFC on Suppression	-0.068	0.027	-2.519	.012	-0.121	-0.016	-0.188
Self-Control on RPE	-0.055	0.029	-1.910	.056	-0.112	0.001	-0.137
Reappraisal on RPE	-0.093	0.034	-2.707	.007	-0.156	-0.020	-0.177
Suppression on RPE	0.011	0.051	0.209	.834	-0.089	0.111	0.016
NFC on RPE	-0.039	0.020	-1.994	.046	-0.076	0.000	-0.160
Indirect Effects							
NFC on RPE via Self-Control	-0.007	0.005	-1.403	.161	-0.019	0.000	-0.030
NFC on RPE via Reappraisal	-0.005	0.004	-1.217	.224	-0.014	0.002	-0.020
NFC on RPE via Suppression	0.001	0.004	-0.191	.848	-0.009	0.006	-0.003
Total Effect							
Total Effect	-0.052	0.021	-2.518	.012	-0.090	-0.010	-0.212

Note: *B* = unstandardized regression coefficient, *β* = standardized regression coefficient, CI = confidence interval, NFC = Need for Cognition, RPE = reduced personal efficacy subscale of theMaslach Burnout Inventory, *SE* = standard error, *N* = 179.

The baseline model did not fit the data ($\chi^2(10, N = 179) = 36.89, p < .001$). Applying the cutoffs by Hu and Bentler (1999) to the fit indices of *CFI* = 1.00, *TLI* = 1.17, *SRMR* = 0.02, and *RMSEA* = 0.00, 95% *CI* [0.00,0.10], suggested good fit of the proposed model throughout all indices.

Table S2.3
Results of the demand-resource-ratio model.

Path	*B*	*SE*	*z*-value	*p*-value	CI Lower	CI Upper	*β*
Direct Effects							
NFC on DTH	-0.035	0.020	-1.789	.074	-0.074	0.003	-0.183
NFC on DTL	-0.020	0.015	-1.287	.198	-0.050	0.010	-0.152
NFC on DRF	0.060	0.020	2.942	.003	0.020	0.100	0.318
NFC on MBI	0.024	0.151	0.161	.872	-0.272	0.320	0.010
DTH on MBI	11.464	2.117	5.416	.000	7.316	15.612	0.912
DTL on MBI	1.951	1.565	1.247	.212	-1.115	5.018	0.106
DRF on MBI	-3.565	1.020	-3.495	.000	-5.564	-1.566	-0.280
Indirect Effects							
NFC on MBI via DTH	-0.403	0.230	-1.754	.079	-0.853	0.047	-0.167
NFC on MBI via DTL	-0.039	0.034	-1.134	.257	-0.106	0.028	-0.016
NFC on MBI via DRF	-0.215	0.104	-2.070	.038	-0.418	-0.011	-0.089
Total Effect							
Total Effect	-0.632	0.253	-2.498	.012	-1.128	-0.136	-0.262

Note: *B* = unstandardized regression coefficient, *β* = standardized regression coefficient, CI = confidence interval, DTH = Demands Too High, DTL = Demands Too Low, DRF = Demand Resource Fit, MBI = Maslach Burnout Inventory, NFC = Need for Cognition, *SE* = standard error, *N* = 179.

The robust Chi-square statistic of $\chi 2(97, N = 179) = 376.01$ ($p < .001$) did not indicate good model fit. However, since it was in the range of 4 df < χ2 > 5 df the lack of good fit might have been due to the underlying assumption of multivariate normality (Hu and Bentler, 1999; Schumacker and Lomax, 2012), which was violated here. This also held true for the CFI of 0.77, the SRMR of 0.17, and the RMSEA of 0.13, 90% CI [0.11,0.14]. Overall, the fit indices did not support the proposed model.

S3: Replication of Grass et al. (2018) when including years spent teaching

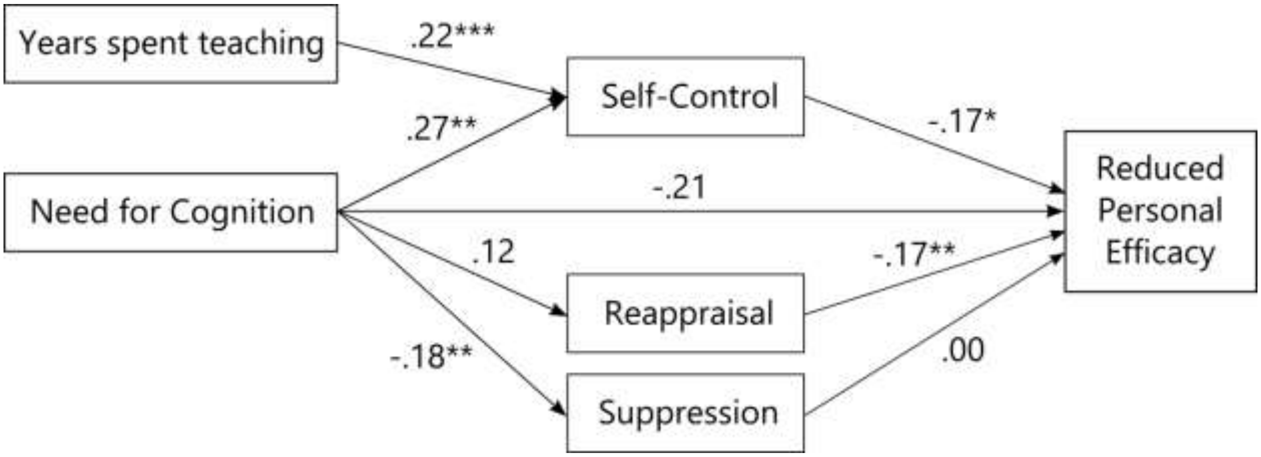

Figure S3.1. Standardized regression coefficients in the replication of Grass et al. (2018) when including years spent teaching. * p < .05, ** p < .01, ** p < .001, N = 180.

Table S3.2

Results of the replication of Grass et al. (2018) when including years spent teaching.

Path	*B*	*SE*	*z*-value	*p*-value	CI Lower	CI Upper	*β*
Direct Effects							
NFC on Self-Control	0.168	0.052	3.258	.001	0.064	0.267	0.280
Years spent teaching on Self-Control	0.145	0.044	3.299	.001	0.054	0.230	0.223
NFC on Reappraisal	0.055	0.036	1.519	.129	-0.016	0.125	0.123
NFC on Suppression	-0.063	0.024	-2.602	.009	-0.109	-0.014	-0.182
Self-Control on RPE	-0.069	0.030	-2.271	.023	-0.127	-0.010	-0.169
Reappraisal on RPE	-0.094	0.036	-2.618	.009	-0.164	-0.022	-0.173
Suppression on RPE	0.002	0.049	0.044	.965	-0.093	0.101	0.003
NFC on RPE	-0.051	0.020	-2.491	.013	-0.089	-0.010	-0.208
Indirect Effects							
NFC and Years spent teaching on RPE via Self-Control	-0.021	0.011	-1.965	.049	-0.045	-0.002	-0.085
NFC on RPE via Reappraisal	-0.005	0.004	-1.325	.185	-0.014	0.002	-0.021
NFC on RPE via Suppression	0.000	0.003	-0.041	.968	-0.008	0.006	-0.001
Total Effect							
Total Effect	-0.078	0.025	-3.164	.002	-0.124	-0.027	-0.315

Note: *B* = unstandardized regression coefficient, *β* = standardized regression coefficient, CI = confidence interval, NFC = Need for Cognition, RPE = reduced personal efficacy subscale of the Maslach Burnout Inventory, *SE* = standard error, *N* = 180.

S4: Demand-resource-ratio model with the MBI subscale reduced personalefficacy

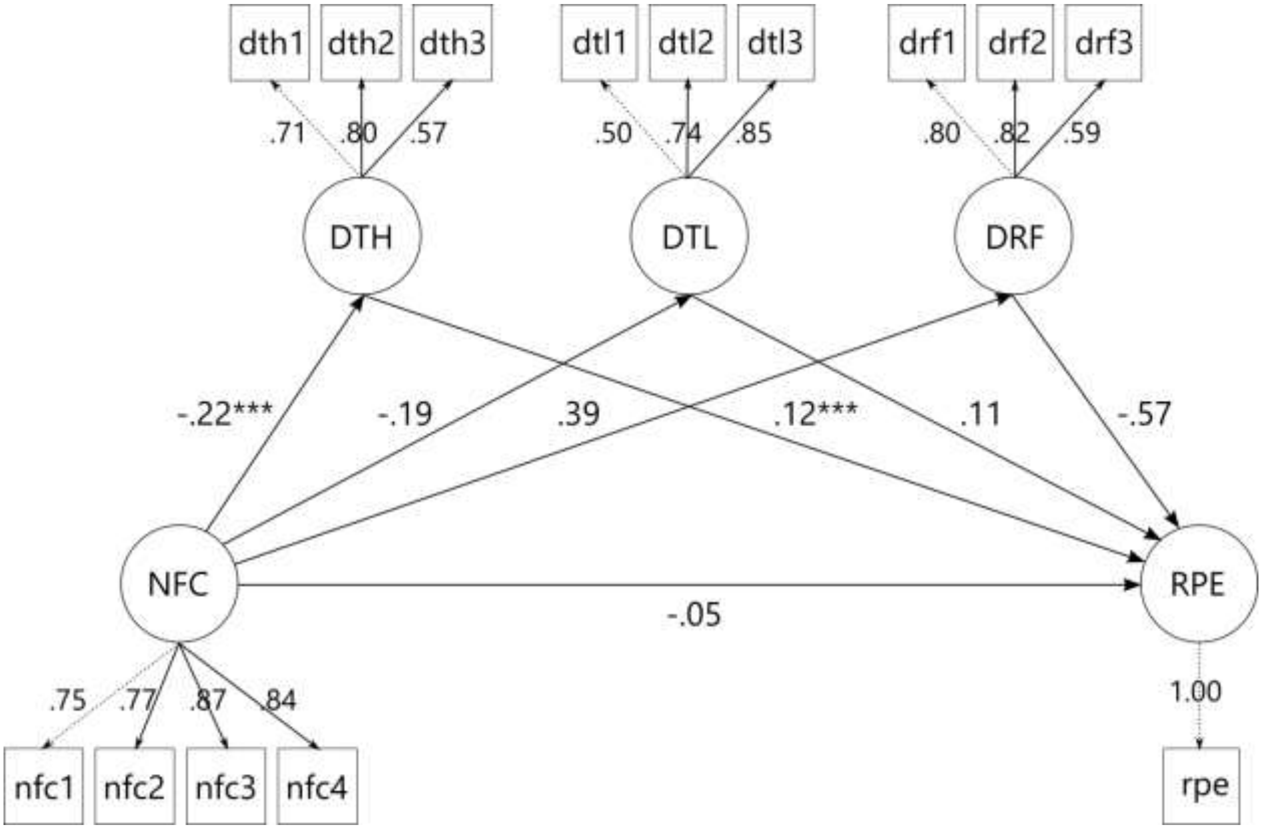

Figure S4.1. Standardized path coefficients of the demand-resource-ratio model with the MBI subscale reduced personal efficacy. * $p < .05$, ** $p < .01$, *** $p < .001$. NFC = Need for Cognition, DTH = demands too high, DTL = demands too low, DRF = demand resource fit, nfc1-4 = item parcels, dth/dtl/drf1-3 = item indicators, RPE = reduced personal efficacy, N = 180.

S5: Exploratory model with all relevant variables

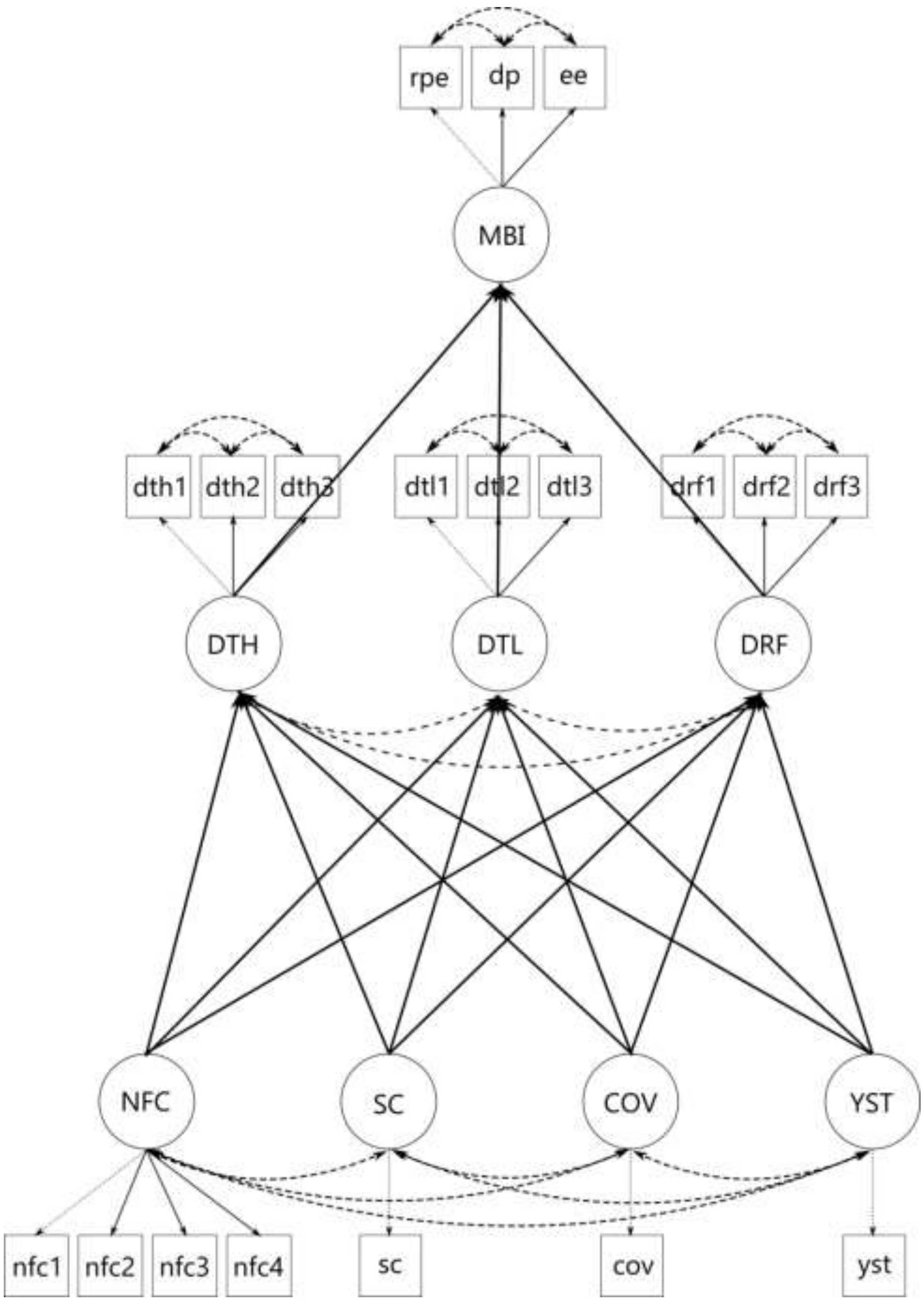

Figure S5.1. Theorized full model with all variables (without emotion regulation). NFC = Need for Cognition, DTH = demands too high, DTL = demands too low, DRF = demand resource fit, MBI = Maslach Burnout Inventory, NFC = Need for Cognition, DTH = Demands Too High, DTL = Demands Too Low, DRF = Demand-Resource-Fit, nfc1-4 = item parcels, dth/dtl/drf1-3 = item indicators, ee = emotional exhaustion, dp = depersonalisation, rpe = reduced personal efficacy, sc/SC = Self-Control Scale, cov/COV = Covid-19 burden, yst/YST = years spent teaching.

Table S5.2
Results of the exploratory model with Covid burden.

Path	*B*	*SE*	*z*-value	*p*-value	CI Lower	CI Upper	*β*
Direct Effects							
YST on COVB	0.055	0.024	2.327	.020	0.009	0.102	0.168
Years on SCS	0.137	0.045	3.037	.002	0.049	0.226	0.212
COVB on DTH	0.061	0.014	4.352	.000	0.034	0.089	0.449
SCS on DTH	-0.015	0.005	-3.069	.002	-0.025	-0.005	-0.217
NFC on DTH	-0.038	0.014	-2.646	.008	-0.065	-0.010	-0.210
SCS on DRF	0.015	0.006	2.540	.011	0.003	0.026	0.223
NFC on DRF	0.057	0.018	3.162	.002	0.022	0.093	0.336
DTH on EE	14.985	2.111	7.098	.000	10.847	19.124	1.004
COVB on EE	-0.294	0.136	-2.161	.031	-0.560	-0.027	-0.144
DRF on RPE	-4.686	0.634	-7.387	.000	-5.930	-3.443	-0.760
Indirect Effects							
NFC and YST on RPE via SCS and DRF	-0.279	0.084	-3.319	.001	-0.443	-0.114	-0.291
NFC and YST on EE via SCS, COVB, and DTH	-0.543	0.206	-2.633	.008	-0.947	-0.139	-0.181
Total Effect							
Total Effect	-0.821	0.256	-3.212	.001	-1.322	-0.320	-0.472

Note: *B* = unstandardized regression coefficient, *beta* = standardized regression coefficient, CI = confidence interval, COVB = Covid Burden, DTH = Demands Too High, DRF = Demand Resource Fit, MBI = Maslach Burnout Inventory, NFC = Need for Cognition, SCS = Self-controlScale, *SE* = standard error, YST = Years spent teaching, *N* = 180.

S6: Replication of Grass et a. (2018) and Demand-Resource-Ratio Model with the emotional exhaustion and depersonalization subscales.

Table S6.1

Results of the replication of Grass et al. (2018), including years spent teaching, with the emotional exhaustion subscale.

Path	*B*	*SE*	*z*-value	*p*-value	CI Lower	CI Upper	*β*
Direct Effects							
NFC on Self-Control	0.168	0.050	3.363	.001	0.063	0.261	0.280
Years spent teaching on Self-Control	0.145	0.044	3.320	.001	0.058	0.226	0.223
NFC on Reappraisal	0.055	0.034	1.619	.105	-0.011	0.120	0.123
NFC on Suppression	-0.063	0.025	-2.524	.012	-0.113	-0.017	-0.182
Self-Control on EE	-0.263	0.070	-3.756	.000	-0.397	-0.118	-0.250
Reappraisal on EE	-0.072	0.107	-0.674	.500	-0.286	0.135	-0.051
Suppression on EE	-0.109	0.141	-0.773	.440	-0.379	0.170	-0.060
NFC on EE	-0.095	0.052	-1.848	.065	-0.193	0.005	-0.151
Indirect Effects							
NFC and Years spent teaching on EE via Self-Control	-0.082	0.029	-2.867	.004	-0.141	-0.031	-0.126
NFC on EE via Reappraisal	-0.004	0.007	-0.565	.572	-0.020	0.009	-0.006
NFC on EE via Suppression	0.007	0.010	0.718	.473	-0.012	0.027	0.011
Total Effect							
Total Effect	-0.175	0.055	-3.162	.002	-0.279	-0.064	-0.272

Note: *B* = unstandardized regression coefficient, *β* = standardized regression coefficient, CI = confidence interval, NFC = Need for Cognition, EE = emotional exhaustion subscale of the Maslach Burnout Inventory, *SE* = standard error, *N* = 180.

$\chi 2(6, N = 180) = 2.70$ (p = .845)

CFI = 1.00, RMSEA = 0.00, 90% CI [0.00,0.06], SRMR = 0.03

Table S6.2

Results of the replication of Grass et al. (2018), including years spent teaching, with the depersonalization subscale.

Path	*B*	*SE*	*z*-value	*p*-value	CI Lower	CI Upper	*β*
Direct Effects							
NFC on Self-Control	0.168	0.050	3.327	.001	0.067	0.264	0.280
Years spent teaching on Self-Control	0.145	0.045	3.212	.001	0.055	0.232	0.223
NFC on Reappraisal	0.055	0.036	1.516	.130	-0.018	0.126	0.123
NFC on Suppression	-0.063	0.026	-2.454	.014	-0.111	-0.013	-0.182
Self-Control on DP	-0.122	0.027	-4.485	.000	-0.174	-0.069	-0.316
Reappraisal on DP	-0.017	0.032	-0.522	.601	-0.075	0.051	-0.033
Suppression on DP	0.019	0.048	0.408	.683	-0.072	0.118	0.029
NFC on DP	-0.041	0.020	-2.044	.041	-0.079	0.000	-0.175
Indirect Effects							
NFC and Years spent teaching on DP via Self-Control	-0.038	0.012	-3.182	.001	-0.063	-0.017	-0.159
NFC on DP via Reappraisal	-0.001	0.002	-0.428	.668	-0.005	0.003	-0.004
NFC on DP via Suppression	-0.001	0.003	-0.376	.707	-0.009	0.005	-0.005
Total Effect							
Total Effect	-0.081	0.025	-3.213	.001	-0.130	-0.029	-0.344

Note: *B* = unstandardized regression coefficient, *β* = standardized regression coefficient, CI = confidence interval, NFC = Need for Cognition, DP = depersonalization subscale of the Maslach Burnout Inventory, *SE* = standard error, *N* = 180.

$\chi 2(6, N = 180) = 5.98$ (p = .426)

CFI = 1.00, RMSEA = 0.00, 90% CI [0.00,0.10], SRMR = 0.04

Table S6.3

Results of the demand-resource-ratio model with the emotional exhaustion subscale.

Path	*B*	*SE*	*z*-value	*p*-value	CI Lower	CI Upper	β
Direct Effects							
NFC on DTH	-0.041	0.019	-2.176	.030	-0.078	-0.004	-0.224
NFC on DTL	-0.023	0.015	-1.480	.139	-0.053	0.007	-0.179
NFC on DRF	0.069	0.020	3.442	.001	0.030	0.108	0.386
NFC on EE	0.059	0.148	0.399	.690	-0.230	0.348	0.023
DTH on EE	12.353	1.930	6.402	.000	8.571	16.135	0.869
DTL on EE	1.415	1.550	0.913	.361	-1.622	4.452	0.069
DRF on EE	-2.494	0.878	-2.840	.005	-4.215	-0.772	-0.170
Indirect Effects							
NFC on EE via DTH	-0.507	0.231	-2.192	.028	-0.961	-0.054	-0.194
NFC on EE via DTL	-0.032	0.031	-1.048	.295	-0.093	0.028	-0.012
NFC on EE via DRF	-0.171	0.086	-1.983	.047	-0.340	-0.002	-0.066
Total Effect							
Total Effect	-0.652	0.242	-2.699	.007	-1.126	-0.179	-0.250

Note: *B* = unstandardized regression coefficient, β = standardized regression coefficient, CI = confidence interval, DTH = Demands Too High, DTL = Demands Too Low, DRF = Demand Resource Fit, EE = emotional exhaustion subscale of the Maslach Burnout Inventory, NFC = Need for Cognition, *SE* = standard error, *N* = 180.

Robust $\chi2(71, N = 180) = 287.90$ ($p < .001$)

Robust CFI = 0.82, RMSEA = 0.13, 90% CI [0.12,0.15], SRMR = 0.17

Table S6.4

Results of the demand-resource-ratio model with the depersonalization subscale.

Path	*B*	*SE*	*z*-value	*p*-value	CI Lower	CI Upper	*β*
Direct Effects							
NFC on DTH	-0.054	0.024	-2.228	.026	-0.102	-0.007	-0.218
NFC on DTL	-0.024	0.015	-1.637	.102	-0.054	0.005	-0.187
NFC on DRF	0.069	0.019	3.572	.000	0.031	0.107	0.385
NFC on DP	-0.091	0.080	-1.145	.252	-0.248	0.065	-0.094
DTH on DP	0.692	0.331	2.089	.037	0.043	1.341	0.176
DTL on DP	1.240	0.812	1.528	.127	-0.351	2.831	0.166
DRF on DP	-1.912	0.507	-3.769	.000	-2.906	-0.918	-0.352
Indirect Effects							
NFC on DP via DTH	-0.037	0.025	-1.509	.131	-0.086	0.011	-0.039
NFC on DP via DTL	-0.030	0.024	-1.255	.210	-0.078	0.017	-0.031
NFC on DP via DRF	-0.132	0.053	-2.504	.012	-0.235	-0.029	-0.135
Total Effect							
Total Effect	-0.291	0.104	-2.788	.005	-0.495	-0.086	-0.299

Note: *B* = unstandardized regression coefficient, *β* = standardized regression coefficient, CI = confidence interval, DTH = Demands Too High, DTL = Demands Too Low, DRF = Demand Resource Fit, DP = depersonalization subscale of the Maslach Burnout Inventory, NFC = Need for Cognition, *SE* = standard error, *N* = 180.

Robust $\chi 2(71, N = 180) = 245.31$ ($p < .001$)

Robust CFI = 0.83, RMSEA = 0.12, 90% CI [0.10,0.13], SRMR = 0.16
